# Dimerization and lysine substitution of melittin have differing effects on bacteria

**DOI:** 10.3389/fphar.2024.1443497

**Published:** 2024-10-07

**Authors:** Tamara Matthyssen, Wenyi Li, James A. Holden, Jason C. Lenzo, Sara Hadjigol, Neil M. O’Brien-Simpson

**Affiliations:** ^1^ ACTV Research Group, Melbourne Dental School, Division of Basic and Clinical Oral Sciences, Royal Dental Hospital and The Bio21 Institute of Molecular Science and Biotechnology, The University of Melbourne, Melbourne, VIC, Australia; ^2^ Department of Biochemistry and Chemistry, La Trobe Institute for Molecular Science, La Trobe University, Melbourne, VIC, Australia; ^3^ Melbourne Dental School, Centre for Oral Health Research, Royal Dental Hospital and The Bio21 Institute of Molecular Science and Biotechnology, The University of Melbourne, Melbourne, VIC, Australia; ^4^ Western Australian Health Translation Network, Harry Perkins Institute of Medical Research, Nedlands, WA, Australia

**Keywords:** peptide, antimicrobial activity, melittin, melittin analog, toxicity

## Abstract

**Introduction:**

Melittin is a potent antimicrobial peptide from bee venom that is effective against both Gram-positive and Gram-negative bacteria. However, it is extremely toxic to mammalian cells and, as yet, has no clinical use. Modifications to its amino acid sequence, cyclization, truncation, and dimerization have been attempted in order to reduce its toxicity whilst maintaining its antimicrobial activity.

**Methods:**

In this study, we targeted the three lysine residues present in melittin and substituted them with lysine homologs containing shorter side chains (ornithine, Orn, diaminobutyric acid, Dab, and diaminopropanoic acid, Dap) and made both parallel and antiparallel melittin dimers to observe how lysine substitution and dimerization affects its activity and toxicity. The antibacterial activity of melittin and its analogs was tested against *S. aureus* (Gram-positive bacteria) and *E. coli* (Gram-negative bacteria), and cytotoxicity was tested against the mammalian cell lines HEK293 and H4IIE.

**Results:**

Overall, dimerization and lysine substitution exhibited improved antimicrobial activity toward *E. coli* and limited improvement toward *S. aureus.* However, mammalian cell toxicity was only marginally reduced compared to native melittin. Interestingly, the parallel dimer was found to be marginally more active than the antiparallel dimer, indicating orientation maybe important for activity, although both dimers were less effective than the native and Lys-analog peptides toward *S. aureus*. Of the Lys substitutions, Dab and Dap improved melittin’s activity toward *E. coli*.

**Discussion:**

Dimerization and Lys substitution of melittin improved the antimicrobial activity toward Gram-negative bacteria but did not significantly improve its activity toward Gram-positive bacteria. Some analogs also displayed reduced toxicity toward HEK293 and H4IIE cells but overall remained toxic at bactericidal concentrations. Our data indicates that although highly antibacterial, melittin’s toxicity is the major drawback in its potential use.

## Introduction

Infections caused by drug-resistant bacteria have become an increasing threat to public health, with an estimated 4.95 million deaths associated with antimicrobial resistance (AMR) in 2019 (Murray, 2022). It is estimated that six pathogens were the leading cause of AMR-associated deaths: *Escherichia Coli*, *Staphylococcus aureus*, *Klebsiella pneumoniae*, *Streptococcus pneumoniae*, *Acinetobacter baumannii*, and *Pseudomonas aeruginosa*. Moreover, this rise in AMR-related deaths has been accompanied by a decrease in the development of novel antimicrobials (Brown and Wright, 2016). In a bid to combat resistant organisms and to not induce resistance or slow its development, research has focused on antimicrobial peptides (AMPs). Found in various organisms, AMPs exhibit strong antimicrobial activity against a wide variety of both Gram-positive and Gram-negative bacteria (including drug-resistant strains) as well as fungi, viruses, and parasites (Hancock and Sahl, 2006; Hancock and Lehrer, 1998; Zasloff, 2002; Ageitos et al., 2017). However, when used in an exogenous manner, their cell selectivity is greatly diminished, and these promising therapeutics can also be toxic to host cells. A recent review by Torres et al. highlighted amino acid substitution, the hydrophobic/hydrophilic ratio of amino acids, the net charge, overall structure, and the addition of unnatural modifications as key areas to target to improve the design process and cell selectivity of AMPs (Torres et al., 2019). AMP development is one avenue being pursued for the discovery of new therapeutics that can combat the threat of multidrug-resistant bacteria and the emergence of “super-bugs.”

**FIGURE 1:**
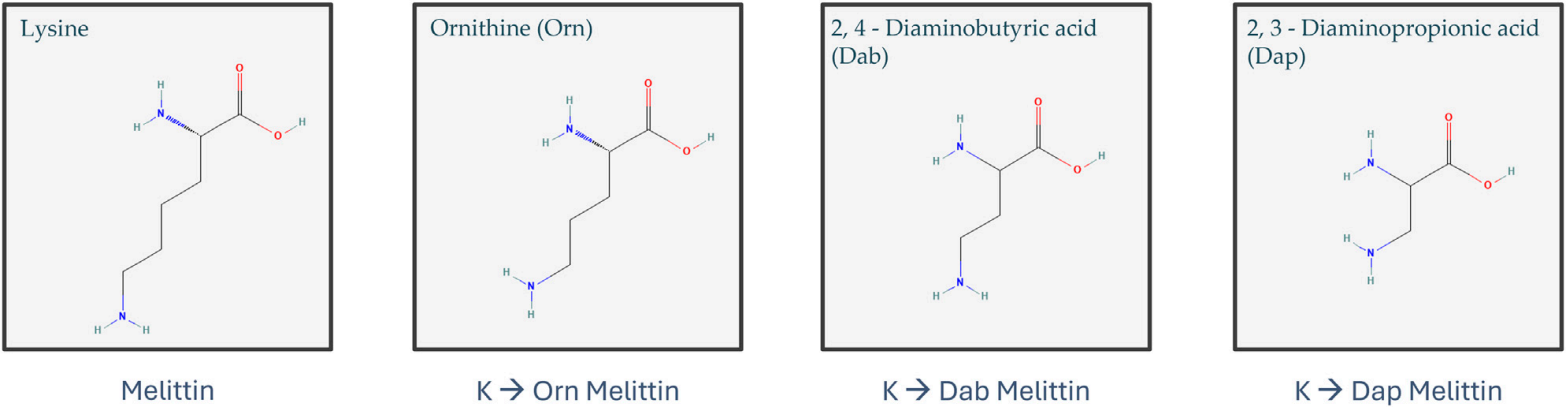
Structure of lysine, ornithine (Orn), 2,4-diaminobutyric acid (Dab), and 2,3-diaminopropionic acid (Dap) (structures obtained from PubChem (https://pubchem.ncbi.nlm.nih.gov/)) (Kim et al., 2023; National Center for Biotechnology Information, 2024a, 2024b, 2024c, 2024d).

Melittin is a well-known AMP from European honeybee venom that is highly efficacious at eradicating bacteria but is also extremely toxic to mammalian cells due to its general mode of action of membrane permeabilization (Askari et al., 2021; Memariani et al., 2019). Given that melittin’s inherent purpose in nature is to protect its host from a variety of predators, it is unsurprising that there is little selectivity in the types of membranes it can permeabilize, and thus, this toxicity is a major barrier to its clinical development (Guha et al., 2021). Many attempts have been made to modify melittin to reduce its toxicity whilst maintaining its activity. These modifications have included the substitution of specific amino acids (such as proline and leucine), cyclization, truncation, and dimerization of truncated melittin (Rex, 2000; Krauson et al., 2015; Mayandi et al., 2020; Pandey et al., 2010; Unger et al., 2001). Here, we attempted to reduce the toxicity and improve the antimicrobial activity of melittin by targeting its structure and amino acid composition through either dimerization or substitution of the three lysine residues present at positions 7, 21, and 23 with lysine mimetic derivatives (Figure 1). Previous work has shown that replacing Lys residues with lysine mimetic derivatives (ornithine (Orn), 2, 4-diaminobutyric acid (Dab) or 2, 3-diaminopropionic acid (Dap)) resulted in reduced toxicity of an AMP, whereas dimerization improved antimicrobial activity (Li W. et al., 2017; O’Brien-Simpson et al., 2016). Mayandi et al. also showed that the substitution of melittin’s α-lysines with ε-lysine improved activity and reduced toxicity (Mayandi et al., 2020). In a separate study, Tran et al. demonstrated that substitution of lysine in an engineered stapled heptapeptide with Orn, Dab, or Dap reduced hemolysis, had varying effects on its activity toward a variety of Gram-positive and Gram-negative bacteria, and increased their proteolytic stability (Tran et al., 2024). Substitution with Orn, Dab, and Dap does not alter the overall charge of an AMP, but these amino acids do differ in the length of their side chains and may give insights into the subtle features of lysine that make it indispensable to many AMPs (Figure 1). In this study, we were interested in observing how different peptide modifications affect typical Gram-positive and Gram-negative bacteria and whether they impact different membranes in different ways. As such, *S. aureus* and *E. coli* were chosen as model Gram-positive and Gram-negative bacteria, respectively, as they are widely used in many AMP studies and are on the WHO list of pathogens that are the leading cause of AMR-associated deaths (World-Health-Organisation, 2017). This work offers insights into the role that dimerization and reduced side-chain length of lysine residues play in the activity of melittin against a Gram-positive *versus* Gram-negative bacteria and highlights the extreme cytotoxicity of full-length melittin as a major barrier to its clinical use as an antimicrobial.

## Materials and methods

### Determining CFUs/mL

The bacterial strains *S. aureus* ATCC 29213 and *E. coli* ATCC 8739 lyophilized or glycerol stocks were obtained from the culture collection of The Melbourne Dental School, University of Melbourne, Australia. These strains were selected for this study as they are widely used in antimicrobial studies and are considered to be important pathogens and major targets for antibiotic-resistant studies. The bacteria were grown aerobically and maintained on Lysogeny/Luria Broth (LB) agar (1% w/v Oxoid tryptone, 0.5% w/v NaCl, 0.5% w/v Oxoid Yeast Extract and 1.5% w/v BD Bacto™ Agar, Thermo Scientific Pty, Ltd., Sydney, Australia, pH 7.0) at 4°C. Growth curves and colony forming units (CFUs) per mL were determined as follows: for each bacterium, one colony from an LB agar plate was suspended in 10 mL LB broth and grown aerobically at 37°C in an orbital shaker (Minitron, Infors HT) at 200 rpm. After overnight incubation, 95 mL of fresh LB broth was inoculated with 5 mL of the overnight culture so that the final solution was a 5% v/v inoculum. The optical density (OD) was measured every 30 min at 600 nm using a spectrophotometer (model Varian Cary 50 Bio UV-Vis) and plotted against time to generate a growth curve. Each sample was then titrated out and spotted onto LB agar plates. In brief, 20 µL of the bacteria culture at each time point was titrated out across eight wells in phosphate-buffered saline (PBS, 137 mM NaCl, 2.7 mM KCl, 10 mM Na_2_HPO_4_, and 1.80 mM KH_2_PO_4_). From each well, 10 µL was spotted onto an LB agar plate and incubated overnight at 37°C. The next day, the CFUs were counted, and CFUs/mL were determined. The CFUs/mL were then plotted against the OD_600_ reading of the sample, and a line of best fit was determined. The equation of the line of best fit was later used to determine CFUs/mL according to the OD_600_.

### Synthesis of antimicrobial peptides (AMPs)

2-Cyano-2-(hydroxyamino) acetate (Oxyma), *N*,*N′*-diisopropylcarbodiimide (DIC), and 9-fluorenylmethoxycarbonyl (Fmoc) amino acids were obtained from Mimotopes Pty Ltd. (Melbourne, Australia). Triisopropylsilane (TIPS), *N*,*N*-dimethylformamide (DMF), piperidine, trifluoroacetic acid (TFA), diethyl ether, and dichloromethane (DCM) were obtained from Aldrich (New South Wales, Australia). Unless otherwise stated, all the chemicals were of analytical grade or equivalent and used directly without further treatment.

AMPs and peptides for multimerization (Table 1) were chemically synthesized on a CEM Liberty-blue microwave peptide synthesizer (Ai Scientific, Victoria, Australia). The peptide-resins were assembled on Fmoc-Rink-Amide AM SURE™ resin (0.68 mmol/g) to produce the C-terminal carboxamide peptides. For a 0.1 mmol reaction scale, Fmoc-deprotection was performed in 20% piperidine/DMF (v/v) under microwave radiation for 15 s (155 W, 75°C), followed by filtration and a second addition of the above solution (30 W, 90°C; 50 s). The peptide-resins were then rinsed with DMF (4 × 7 mL). Standard Fmoc (9-fluorenylmethoxy carbonyl) chemistry was used throughout with a 5-fold molar excess of Fmoc-protected amino acids in the presence of Oxyma (5 eq) and DIC (10 eq) in DMF/DCM (1:1, v/v; 4 mL) added to the Nα-amino deprotected peptide-resin. The mixture was coupled under microwave radiation for 15 s (170 W, 75°C), followed by another 110 s (30 W, 90°C). The addition of underlined amino acids in Table 1 was accomplished by double coupling. For the melittin dimers, the parallel dimer was synthesized by simultaneously synthesizing both arms from the C to N terminus, one from the amino group on the α carbon and the other from the side-chain amino group of 2,4-diaminobutyric acid (Dab). The antiparallel melittin dimer was synthesized first from the C to the N terminus, Boc-Lys (Fmoc) was added, and the synthesis continued from the C to the N terminus of the second melittin from the amino group on the ε carbon of the Lys.

**TABLE 1:** Peptides and their amino acid sequences.

Melittin variation	Amino acid sequence
1. Melittin	GIGAVLKVLTTGLPALISWIKRKRQQ-CONH_2_
2. Lys → Orn melittin	GIGAVL (Orn)VLTTGLPALISWI(Orn)R (Orn)RQQ-CONH_2_
3. Lys → Dab melittin	GIGAVL (Dab)VLTTGLPALISWI(Dab)R (Dab)RQQ-CONH_2_
4. Lys → Dap melittin	GIGAVL (Dap)VLTTGLPALISWI(Dap)R (Dap)RQQ-CONH_2_
5. Parallel melittin dimer	GIGAVLKVLTTGLPALISWIKRKRQQ 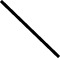 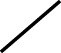 Dab-CONH_2_ GIGAVLKVLTTGLPALISWIKRKRQQ
6. Antiparallel melittin dimer	QQRKRKIWSILAPLGTTLVKLVAGIG 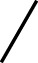 K-GIGAVLKVLTTGLPALISWIKRKRQQ-CONH2

Underlined amino acids indicate double coupling.

Orn, ornithine; Dab, 2,4-diaminobutyric acid; Dap, 2,3-diaminopropionic acid

Post synthesis, the peptide-bound resin was washed with DCM (5 × 10 mL) and diethyl ether (5 × 10 mL) and allowed to dry prior to the peptide cleavage. The peptides were cleaved from the resin support by the addition of TFA/TIPS/phenol/water (95:3:1:1, % v/v/v/v; 10 mL) for 2.5 h or 4 h for Arg-containing peptides, under nitrogen and no light. Following cleavage, the TFA/peptide solution was isolated by filtration, the volume was reduced (1 mL) under nitrogen, and the crude product was isolated by precipitation in cold ether (4 × 20 mL). The dried crude peptides were then dissolved in buffer B (0.1% TFA in 90% acetonitrile, 10% milliQ water, v/v) and buffer A (0.1% v/v TFA in milliQ water) added so the final concentration of acetonitrile was 10%. The crude peptides were purified using a semi-preparative ZORBAX 300 SB-C18 column (9.4 mm × 25 cm) installed in a High Performance Liquid Chromatography (HPLC) 1,200 system (Agilent Technologies Pty. Ltd., VIC, Australia) under a flow rate of 2 mL/min using buffer A (0.1% v/v TFA in milliQ water) and buffer B (0.1% TFA in 90% acetonitrile, 10% milliQ water, v/v) as the limiting solvent. Peptide detection was performed by absorbance at 214 nm. Fractions were collected and identified using an Exactive™ OrbiTrap Mass Spectrometer (Thermo Fisher Scientific). Purified peptides (greater than 99% purity) were lyophilized, and peptide-TFA salt was displaced by three rounds of lyophilization in 5 mM HCl to form the peptide-HCl salt and then stored at −20°C (Sani et al., 2007).

### Growth conditions for antimicrobial assays

For the antimicrobial peptide assays, a 10 mL starter culture was produced by taking single colonies from LB agar plates to inoculate in Lysogeny/Luria Broth (1% w/v Oxoid Tryptone, 0.5% w/v NaCl, 0.5% w/v Oxoid Yeast Extract, pH 7.0, Thermo Scientific Pty, Ltd., Sydney, Australia) for bacteria; *S. aureus* ATCC 29213 and *E. coli* ATCC 8739 were grown aerobically at 37°C in an orbital shaker (Minitron, Infors HT) at 200 rpm. After overnight incubation, 0.5–1.0 mL of the starter culture was used to inoculate fresh LB broth (20 mL), and its growth was monitored at 600 nm using a spectrophotometer (model Varian Cary 50 Bio UV-Vis). Bacteria were harvested during the late exponential growth phase, and CFUs/mL were determined using the growth curve data set that correlated OD_600_ to CFUs/mL.

### Antimicrobial assays

Antibacterial assays were undertaken to determine the minimum inhibitory concentration (MIC), the minimum bactericidal concentration (MBC), and the level of membrane disruption of each of the antimicrobial peptides (AMPs) and multimerized AMPs. For each bacterium, a stock solution (2 × 10^6^ cells/mL) in cation-adjusted Mueller Hinton Broth (MHB) was made, and an aliquot was incubated with AMPs within 15 min from viable cell count and stock preparation. All AMPs were dissolved in 5% dimethylsulfoxide (DMSO) and 1 mg/mL stock solutions prepared in PBS. Serial dilutions with 2 x dilution factor (16.87–0.18 μM) of the AMP in cation-adjusted MHB (100 μL/well) were made immediately prior to the addition of bacteria. The final assay concentration of DMSO was ≤2.5% v/v, which we have previously shown does not affect bacteria viability or susceptibility to AMPs (O’Brien-Simpson et al., 2016). Aliquots (100 μL) of the bacterial stock solution were added to the AMP serial dilutions (final bacterial concentration 1 × 10^5^ cells/well) and incubated at 37°C for 90 min. Bacteria were also incubated in the absence of AMP to serve as a growth control for the assay. After the 90-min incubation period, the antimicrobial activity (MIC and MBC) was determined as follows:

For MIC, the Clinical and Laboratory Standards Institute (CLSI) broth microdilution assay was followed, with the modifications being that due to the rapid action of the AMPs, the assay incubation time was shortened from 16–20 h to 90 min (CLSI, 2018). In brief, after the 90 min incubation, bacterial growth was monitored at 10 min intervals with shaking at each interval over a 20 h period at OD_630_ using a BioTek 800™ TS Absorbance Reader (Millennium Science Pty Ltd. Melbourne, Australia), which incubated the cultures at 37°C. The MIC was calculated using the Lambert and Pearson growth curve analysis method by plotting the relative growth at each peptide concentration compared to maximal growth (determined as the point when bacteria incubated in broth alone entered the stationary phase of growth, 100% growth). The MIC was determined as the lowest peptide concentration (μM) required to completely inhibit the growth of the bacteria, that is, the intersection of the linear curve with the x-axis (Lambert and Pearson, 2000).

The CLSI protocol was followed to determine MBC. After incubation, bacteria were serially diluted (1:10) in PBS, plated on LB agar, and incubated for 12–24 h at 37°C; then, the CFUs were quantified. The MBC was thus determined as the lowest concentration (μM) of AMP resulting in less than one CFU. The data were fitted to an exponential model, and as such, 1 CFU was considered to be a ≥99.9% reduction in the initial colony count after incubation.

To observe membrane disruption, a 50 μL aliquot of the bacteria/AMP was mixed with 100 μL of 0.9% w/v saline containing 0.07% v/v of SYTO 9 (5 mM stock solution) and 0.04% v/v of PI (1.5 mM stock solution). Following incubation, the bacterial cell samples were analyzed using a CytoFLEX LX flow cytometer (Beckman Coulter). The fluorescence from SYTO 9 was measured through a 525/40 nm band-pass filter, the red emission of PI was measured with a 610/20 nm band-pass filter, and a minimum of 10,000 bacterial events were recorded. The % PI + cells values were plotted against each peptide concentration.

### Cytotoxicity toward cells

To determine toxicity toward mammalian cells, 1 × 10^4^ cells/well of HEK-293 (ATCC CRL-1573TM) or H4IIE in a total volume of 200 μL in media (Eagle’s Minimum Essential Medium (EMEM) supplemented with 10% v/v fetal bovine serum, 5% v/v L-glutamine and 5% v/v penicillin/streptomycin) were seeded into 96-well plates and cultured at 37°C in a 5% CO_2_ incubator for 16–20 h. When cells were ≥90% confluent, the 100 µL media was removed and replaced with 50 μL serial dilutions of the AMP in media (final concentration 20–0.5 μM) and cultured at 37°C in a 5% CO_2_ incubator for 90 min. From each well, 50 µL was transferred to a new plate. To the 50 μL, 50 µL of lactate dehydrogenase (LDH) solution (CytoTox 96^®^ Non-Radioactive Cytotoxicity Assay kit, Promega) was added to each well and incubated for a further 30 min at room temperature and protected from light. Cell death was determined by measuring absorbance at 490 nm using a microplate reader (PerkinElmer 1,420 Multilabel Counter VICTOR3). Positive and negative controls for inhibition were taken as cells incubated with lysis solution (CytoTox 96^®^ Non-Radioactive Cytotoxicity Assay kit, Promega) and cells incubated in media alone, respectively. The background absorbance of media alone was subtracted from the sample absorbance, and the percentage of inhibition was calculated using the following formula:

$$\%\text{ Cytotoxicity}=\left[\left(A490\text{ test sample}-A490\text{ negative control}\right)/\left(A490\text{ positive control}-A490\text{ negative control}\right)\right]\times 100.$$

The percentage of lysis was plotted against peptide concentration, and linear regression analysis was used to determine the AMP concentration needed to lyse 50% (LD_50_) of the cells.

### Inhibition of cell proliferation assay

To determine the inhibition of cell proliferation, 20 μL of 3-(4,5-dimethylthiazol-2-yl)-5-(3-carboxymethoxyphenyl)-2-(4-sulfophenyl)-2H-tetrazolium (MTS) solution (CellTiter 96 AQueous Non-Radioactive Cell Proliferation Assay kit, Promega) was added to the remaining 100 µL from the toxicity assay, and the plates were incubated for a further 1 h at 37°C in a 5% CO_2_ incubator protected from light. Cell proliferation was determined by measuring absorbance at 490 nm using a microplate reader (PerkinElmer 1,420 Multilabel Counter VICTOR3). Positive and negative controls for inhibition were taken as cells incubated with lysis solution (CytoTox 96^®^ Non-Radioactive Cytotoxicity Assay kit, Promega) and cells incubated in media alone, respectively. The background absorbance of media alone was subtracted from the samples, and the percentage of inhibition was calculated using the following formula:

$$\%\text{ Inhibition}=\left[\left(A490\text{ test sample}-A490\text{ negative control}\right)/\left(A490\text{ positive control}-A490\text{ negative control}\right)\right]\times 100.$$

The percentage of inhibition was plotted against peptide concentration, and linear regression analysis was used to determine the AMP concentration needed to inhibit 50% (IC_50_) of cell growth.

### Hemolysis assay

Chicken red blood cells (RBCs) in Alsever’s solution (RBCs, Equicell, Victoria, Australia) were diluted 1 in 20 in PBS (pH 7.4), pelleted by centrifugation, and washed three times in PBS (1,000 g, 10 min). The RBCs were counted using a cell counter (Coulter Particle Counter Z series, Beckman Coulter) and diluted to a final concentration of 2 × 10^7^ cells/mL. Aliquots (100 μL) of the RBC solution were seeded into a V-bottomed 96-well plate containing 100 μL of serial dilutions (10–0.08 μM) of the AMP in PBS and incubated in a humidified atmosphere containing 5% CO_2_ at 37°C for 2 h. Following incubation, the RBCs were pelleted in a centrifuge (1,000 g, 10 min), and the amount of hemoglobin released in 100 μL aliquots of the supernatant was determined by measuring the absorbance at 405 nm using a microplate reader (PerkinElmer 1,420 Multilabel Counter VICTOR3). Positive and negative controls for hemolysis were taken as RBC lysed with 1% v/v Triton X-100 and RBC suspension in PBS, respectively. The percentage of hemolysis was calculated using the following formula:

$$\%\text{ Hemolysis }\left[\left(A405\text{ test sample}-A405\text{ negative control}\right)\;/\;\left(A405\text{ positive control}-A405\text{ negative control}\right)\right]x\;100.$$

The percentage hemolysis was plotted against peptide concentration, and linear regression analysis was used to determine the hemolytic concentration needed to lyse 50% (HC_50_) of RBCs.

### Statistical analysis

All data were obtained from at least three biological repeats (except for membrane disruption, for which the data were obtained from two biological repeats) and expressed as mean ± standard deviation. The data were determined to be normally distributed, and equality of variance was assessed using the Brown–Forsythe test. Statistical analyses (one-way ANOVA and multiple paired t-tests) were carried out with Prism v9.0 (GraphPad Software Inc.), and differences were regarded as statistically significant when *p* < 0.05. The effect size was also calculated using the Cambridge effect size calculator spreadsheet (https://www.cem.org/effect-size-calculator). A negative effect size indicates a lower MIC/MBC of the melittin analog than the native melittin, and a positive effect size indicates a larger MIC/MBC than the native melittin.

## Results and discussion

### Peptide synthesis and purification of melittin and its analogs

Structural changes were made to the native AMP melittin by creating both parallel and antiparallel melittin dimers and lysine melittin analogs in which the three Lys residues (Torres et al., 2019) (Lys^7^, Lys^21^ and Lys^23^) were substituted with Orn, Dab, or Dap. All peptides were synthesized using standard solid phase protocols for microwave addition of amino acids and using Oxyma/DIC chemistry for coupling with each amino acid prior to Fmoc removal with 20% piperidine. Once synthesized, melittin and its analogs were purified by RP-HPLC under optimized conditions and identified by mass spectrometry (Table 2; Supplementary Figures S1-S6). The final product was lyophilized (3×) in 0.05 mM HCl to form a peptide-HCl salt and used in antimicrobial assays to determine the effect of the sequence and structural changes.

**TABLE 2:** Synthesized peptides with their amino acid sequences, expected and observed masses, and the amount of peptide obtained.

Melittin variation	Amino acid sequence	Expected mass (Da)^a^	Observed mass (Da)	Peptide yield (mg, (%))
1. Melittin	GIGAVLKVLTTGLPALISWIKRKRQQ-CONH_2_	2846.5	2845.8	15.68 (5.2%)
2. Lys → Orn melittin	GIGAVL (Orn)VLTTGLPALISWI(Orn)R (Orn)RQQ-CONH_2_	2804.5	2803.72	43.2 (15.4%)
3. Lys → Dab melittin	GIGAVL (Dab)VLTTGLPALISWI(Dab)R (Dab)RQQ-CONH_2_	2762.4	2761.70	17.76 (6.4%)
4. Lys → Dap melittin	GIGAVL (Dap)VLTTGLPALISWI(Dap)R (Dap)RQQ-CONH2	2720.3	2719.70	25.2 (9.3%)
5. (Melittin)2-Dab (Parallel dimer)	(GIGAVLKVLTTGLPALISWIKRKRQQ)2-Dab-CONH_2_	5775.1	5775.6	5.52 (0.96%)
6. (Melittin-ε-)K-Melittin (Antiparallel dimer)	QQRKRKIWSILAPLGTTLVKLVAGIG εK-GIGAVLKVLTTGLPALISWIKRKRQQ	5804.2	5803.7	7.2 (1.2%)

^a^ The expected mass was determined by the CEM, Liberty-blue microwave peptide synthesizer software.

Orn, ornithine; Dab, 2,4-diaminobutyric acid; Dap, 2,3-diaminopropionic acid.

All peptides used in antimicrobial assays are peptide-HCl.

### Melittin and its analogs exhibit strong antimicrobial activity

The MICs and MBCs of the six melittin peptides toward *S. aureus* and *E. coli* were determined as previously described (O’Brien-Simpson et al., 2016) using cation-adjusted MHB as the assay media. Vancomycin and gentamycin were used as comparative positive controls for *S. aureus* and *E. coli,* respectively, as they are clinically used antibiotics for treating nosocomial infections caused by these bacteria.

The MIC and MBC values for each peptide toward *S. aureus* and *E. coli* are shown in Table 3. For *S. aureus,* there was no significant difference (*p* > 0.05) in the MIC and MBC values between the control antibiotic vancomycin, peptide 1 (melittin), and peptides 2–4 (Orn-, Dab-, and Dap-melittin). However, both peptides 5 and 6 (parallel and antiparallel melittin dimers) exhibited a significant decrease (d = 5.99; 95% (*p* < 0.05) CI: 0.70, 5.41 and d = 7.22; 95% (*p* < 0.05) CI: 1.42, 7.30 MBC, respectively) in activity compared to native melittin. The MIC of peptide 5 was statistically lower than peptide 6 (3.83 ± 0.71 µM vs 5.18 ± 0.36 µM, respectively, *p* = 0.008). However, there was no significant difference in their bactericidal (MBC) activity (6.31 ± 1.50 µM vs. 7.25 ± 1.20 µM, respectively). Between the two dimers, peptide 6 had the larger positive effect size, indicating that the antiparallel formation was more detrimental to antimicrobial activity than the parallel formation. As for the lysine substituted analogs, although there were no significant differences in activity compared to native melittin, the large negative effect size of peptide 2 (MIC (d = −1.45; 95% (*p* > 0.05) CI: −3.24, 0.36) suggests improved inhibitory activity. Conversely, the large positive effect size of the MIC and MBC values of peptide 4 (d = 1.36; 95% (*p* > 0.05) CI: −0.77, 2.59 and d = 1.32; 95% (*p* > 0.05) CI: −0.32, 3.29, respectively) suggests that substitution with Dap may reduce the antimicrobial activity compared to native melittin. With decreasing side-chain length from lysine to Orn, Dab, and Dap, there was a significant change in the effect size, with Orn producing an increase in activity, and then with decreasing chain length, there was a decrease in the activity of the melittin Lys analogs with peptide 4 having the weakest activity. This trend is similar to an earlier report where replacing lysine in magainin II with Orn, Dab, and Dap resulted in a decrease in activity correlating to shorter side-chain lengths for another Gram-positive bacteria, *Streptococcus mutans*, but no change in activity against the Gram-negative bacterium *Fusobacterium nucleatum* (O’Brien-Simpson et al., 2016).

**TABLE 3:** MIC and MBC values for melittin and its analogs toward *S. aureus* and *E. coli*.

	*S. aureus* (µM)		*E. coli* (µM)	
Peptide	MIC	MBC	MIC	MBC
1. Melittin	1.44 ± 0.11	1.76 ± 0.76	6.89 ± 0.72	8.67 ± 0.93
2. Lys → Orn melittin	1.28 ± 0.06^b-^	1.83 ± 1.21	4.32 ± 0.31^a^ ^,^ ^b-^	7.10 ± 1.68^b-^
3. Lys → Dab melittin	1.47 ± 0.08	1.76 ± 0.92	5.51 ± 0.52^b-^	5.62 ± 1.10^a^ ^,^ ^b-^
4. Lys → Dap melittin	1.59 ± 0.15^b+^	2.76 ± 0.01^b+^	5.62 ± 0.81^b-^	5.64 ± 1.17^a^ ^,^ ^b-^
5. Parallel melittin	3.83 ± 0.71^a^ ^,^ ^b+^	6.31 ± 1.50^a^ ^,^ ^b+^	4.52 ± 0.72^a^ ^,^ ^b-^	4.39 ± 0.54^a^ ^,^ ^b-^
6. Antiparallel melittin	5.18 ± 0.36^a^ ^,^ ^b+^	7.25 ± 1.20^a^ ^,^ ^b+^	6.02 ± 1.22^b-^	5.64 ± 1.08^a^ ^,^ ^b-^
Gentamycin			2.27 ± 0.33	3.3 ± 0.78
Vancomycin	1.80 ± 0.40	1.61 ± 0.44		

^a^ Significantly different (*p* < 0.05) MIC or MBC, respectively, compared to melittin (native) peptide.

^b^ Large effect size (Cohen’s d > 0.8) MIC or MBC, respectively, compared to melittin (native) peptide with b+ indicating a higher and b− indicating a lower MIC or MBC than native melittin, respectively.

When tested against *E. coli*, peptides 1–6 exhibited no significant differences between their MIC and MBC values, indicating that all peptides retained their bactericidal activity. When compared to peptide 1, both peptide 2 and peptide 5 showed significantly lower MIC values (d = −3.29; 95% (*p* < 0.05) CI: −4.81, −0.44 and d = −3.57; 95% (*p* < 0.05) CI: −6.33, −1.06, respectively). Although peptides 3, 4, and 6 did not exhibit significantly different MIC values compared to melittin, they did display large negative effect sizes (peptide 3, d = −1.92; 95% (*p* > 0.05) CI: −3.64, 0.13; peptide 4, d = −1.76; 95% (*p* > 0.05) CI: −3.09, 0.44; peptide 6, d = −0.93; 95% (*p* > 0.05) CI: −2.16, 1.09), indicating that these analogs are trending toward an increase in antimicrobial activity. For peptides 2–4, decreasing side-chain length trended toward decreasing inhibitory activity, as noted when tested against *S. aureus*. Comparing MBC values, both peptides 5 and 6 showed significantly improved bactericidal activity (d = −4.6; 95% (*p* < 0.05) CI: 7.49, −1.49 and d = −3.26; 95% (*p* < 0.05) CI: −4.5, −0.30, respectively) compared to peptide 1 with considerably large effect sizes. Other examples of dimerized AMPs have shown that dimerization at the C-terminal vs the N-terminal can make a difference in activity; no significant differences in bactericidal activity were observed between the two melittin dimers tested here (Lorenzón et al., 2016). However, peptide 5 had a larger negative effect size than peptide 6, suggesting parallel (C-terminal) dimerization to be more effective, corroborating earlier reports (Lorenzón et al., 2016; Dempsey et al., 2003; Yang et al., 2009). Peptides 3 and 4 also displayed significantly improved MBCs (d = −3.28; 95% (*p* < 0.05) CI: 4.48, −0.29; d = −3.26; 95% (*p* < 0.05) CI: −4.35, −0.23, respectively) while peptide 2 displayed a large negative effect size even though its MBC was not significantly different from peptide 1 (d = −1.69; 95% (*p* > 0.05) CI: −2.61, 0.76). The MBC values indicated an opposite trend to the MIC values, with the shorter sidechain length leading to increasing bactericidal activity. Peptide 2 may be better at disrupting the outer membrane of *E. coli* than peptides 3 and 4 but is less effective at penetrating the inner membrane than peptides 3 and 4. The MBC values indicate that dimerization and Lys substitution with Dab or Dap have the potential to improve the bactericidal activity of melittin toward *E. coli*. For such different structural changes, the outcome was the same, with no significant differences in activity toward *E. coli* between peptides 3, 4, 5, and 6. There were also no significant differences between the MIC and MBC values for each peptide toward *S. aureus* or *E*. *coli* (MBC equivalent to MIC), indicating that dimerization and Lys substitution did not affect the bactericidal mode of action of melittin (Levison and Levison, 2009).

**FIGURE 2:**
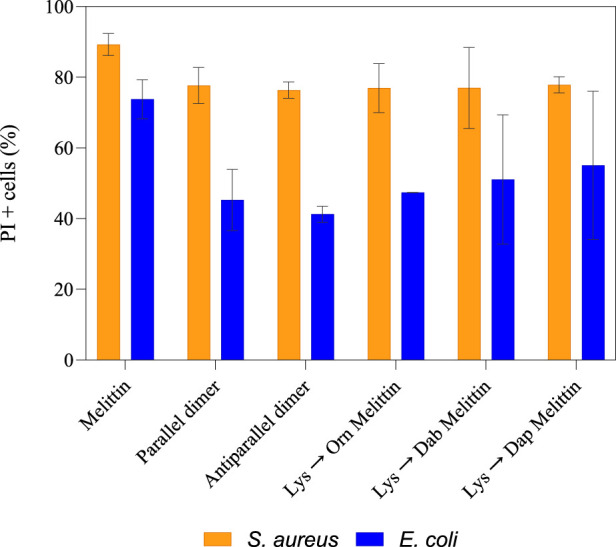
Percentage of PI + cells for *S. aureus* and *E. coli* treated with ∼0.5 × MBC of melittin, melittin dimers, and Lys-substituted melittin. After 90 min incubation of either *S. aureus* or *E. coli* with each peptide at ∼0.5 × MBC, cells were treated with Syto9 and PI and analyzed on a flow cytometer. The percentage of PI + cells was then calculated from two biological replicates. The error bars represent the standard deviation of the sample size.

To observe the cytoplasmic membrane disruption of these peptides, flow cytometry was used to determine the percent PI + cells at ∼0.5 x MBC (O’Brien-Simpson et al., 2016). For *S. aureus*, 89% membrane disruption was observed for melittin, and on average, ∼77% disruption was observed for each analog. For *E. coli*, ∼73% disruption was observed for melittin but only 40%–55% for the melittin dimers and Lys-substituted melittin (Figure 2). Considering both the improved MBC values of the melittin analogs toward *E. coli* and the reduced membrane disruption suggests that the improved activity of the analogs is not only linked to cytoplasmic membrane permeability. However, these differences were not considered significant, and, as such, the dimerization and Lys substitution of melittin did not appear to affect the peptides’ membranolytic mode of action toward *S. aureus* or *E. coli*. There were, however, multiple populations that appeared in the PI + region of *S. aureus* treated with the melittin dimers (Supplementary Figure S7). These populations created a striated appearance, suggesting that the dimers were interacting with each other or the bacterial membrane in a variety of ways and causing multiple degrees of membrane disruption. This striated appearance was also observed to a lesser extent in *E. coli* (Supplementary Figure S8).

Peptides 5 and 6 exhibited decreased antimicrobial activity toward *S. aureus* compared to native melittin but displayed a similar overall level of membrane disruption. Conversely, peptides 3, 4, 5, and 6 showed improved antimicrobial activity toward *E. coli* compared to peptide 1, but there was no significant difference in membrane disruption. The additional outer membrane of *E. coli* can make membrane disruption and, hence, the influx of membrane-impermeable PI, much more difficult. This basic structural difference may explain the difference in PI+ percentages between *S. aureus* and *E. coli*. The lack of difference in membrane disruption of peptide 1 compared to peptides 3, 4, 5, and 6, coupled with their improved MBC values toward *E. coli*, suggests that membrane lysis may not be the only mode of action for these peptides. Although previous research has shown that melittin may act on an internal target, this target has not yet been identified (Ravensdale et al., 2016). Intracellular melittin targets have, however, been identified in human hepatocellular carcinomas (Wu et al., 2015; Liu et al., 2008; Zhang et al., 2016; Zhang et al., 2014). For both *S. aureus* and *E. coli*, the level of membrane disruption did not increase at 1 × MBC.

**FIGURE 3:**
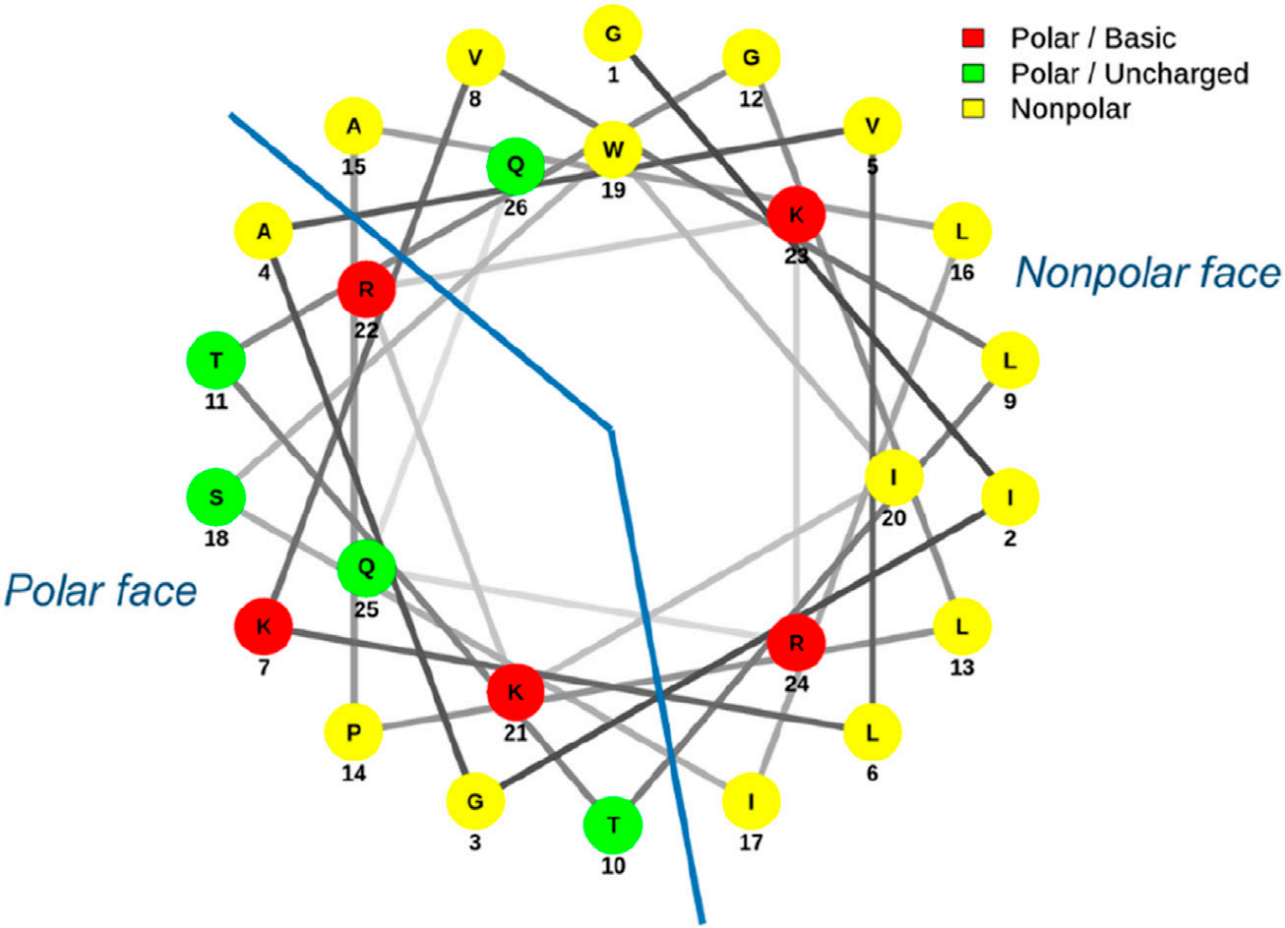
Helical wheel diagram of melittin. Melittin forms an amphipathic structure with most charged residues aligning on the polar face.

Both the parallel and antiparallel dimers have double the amount of melittin for the same molar concentration compared to the other analogs and yet exhibited varying changes in activity depending on which bacteria they were tested against. It is widely accepted that for AMPs to insert into the bacterial membrane, a threshold concentration of peptide at the membrane interface first needs to be achieved; the AMP can only reorient itself and translocate across the membrane to form pores once this threshold concentration has been reached (Hong et al., 2019; Lee et al., 2013). This reasoning has been used to justify, in part, the increased activity observed in many multimerized compounds. However, no increase in activity was observed for either form of dimerized melittin when tested against *S. aureus*; rather, a decrease in activity was noted. Conformational flexibility has been highlighted as important for AMP activity, and in the case of melittin, the flexibility around the Pro residue is important for membrane permeabilization (Krauson et al., 2015). The Pro residue (Pro-14) in the middle of the melittin sequence on the polar face (Figure 3) creates a bend connecting the two α-helices that melittin forms in a hydrophobic environment (Jamasbi et al., 2016; Jeon et al., 2019). It has been shown to be essential for activity as the substitution of Pro with other amino acids significantly reduces the antibacterial activity of melittin (Jamasbi et al., 2014). Computational modeling also suggests melittin may form an intermediate U-shape when inserted into the lipid membrane (Hong et al., 2019). If dimerization affects melittin’s flexibility and adoption of such an intermediary shape, particularly in the presence of peptidoglycan, this may explain the lack of increased activity toward *S. aureus* that is otherwise expected due to the increased and localized peptide concentration.

However, improved bactericidal activity was noted toward *E. coli*. Comparison of the MBC values of each peptide toward *S. aureus* and *E. coli* indicated that the impact on bactericidal activity for both peptides 5 and 6 (parallel and antiparallel dimers) and peptides 3 and 4 (Dab- and Dap-melittin) was bacteria dependent. Dimerization did improve activity toward *E. coli,* as did the substitutions in peptides 3 and 4. This suggests that these modifications may have disrupted/improved specific interactions that occur between melittin and components of the Gram-negative bacterial cell wall. Further studies looking at whether melittin preferentially targets specific bacteria in a mixed model and whether these melittin analogs impact that preference could indicate whether specific interactions with bacterial cell wall components are occurring. Additionally, determining the antimicrobial activity of dimerized Lys-substituted melittin would be of use to determine whether there is a compounding effect of these two strategies.

Melittin’s C-terminus is regarded as highly hydrophilic, with four of its five positively charged amino acids clustered at this end (Figure 3). The positive charge of many AMPs is crucial for their association with negatively charged bacterial membranes and contributes to the selectivity of bacterial cells over eukaryotic cells. With regards to melittin, removal or replacement of the charged residues at its C-terminus does result in reduced affinity for anionic lipid membranes (Hall et al., 2011; Therrien et al., 2016). The overall charge of the Lys-substituted melittins in this study was not changed; however, the parallel and antiparallel dimers had an increased net positive charge. As this increase in charge for the dimers affected the overall antibacterial activity of these peptides in different ways depending on which bacteria it was tested against, it would be interesting to observe the rate at which these dimers assemble on an anionic lipid membrane prior to pore formation. Electrostatic interactions are believed to be the initial step in membrane permeabilization, and observing whether an increased charge facilitates this process would further highlight how changes in structure, and hence charge, impact the sequence of events in AMP action (Li J. et al., 2017). Previous research has indicated that increasing the positive charge only affects antibacterial activity to a certain point, with a charge of +8 to +9 being considered the turning point at which further increases in charge have no effect (Zelezetsky and Tossi, 2006). Melittin and its Lys-substituted analogs have a net charge of +6, whilst the dimers have a net charge of +11. The variation in antimicrobial activity of the dimers compared to the linear analogs suggests that the increased charge of the dimers was not solely responsible for changes in activity; this is consistent with the charge threshold point being approximately +8 to +9 for maximum AMP activity.

Amino acid substitution often results in a change of activity in AMPs, and significant changes were observed in this study when the three Lys residues of melittin were substituted with Orn, Dab, or Dap, and the peptide activity was evaluated against *S. aureus* and *E. coli*. Similar substitutions with these non-proteinogenic amino acids have been reported to also affect the activity of other peptides (magainin II, D16, C18G, and tritrpticin) to different extents (O’Brien-Simpson et al., 2016; Arias et al., 2018; Khara et al., 2016; Kohn et al., 2018; Mant et al., 2019). Such substitutions were capable of either increasing or decreasing activity, and again, this varied depending on which bacteria they were tested against. In these instances, the substitutions may have disrupted or improved features essential to activity, such as overall hydrophobicity and helicity, whereas in melittin, they had no effect. Generally, it is the substitution of hydrophobic amino acids or the substitution of key amino acids (such as the Pro in melittin) with residues that have a different function that results in a change in activity (Jamasbi et al., 2016). This change in activity is often due to changes in amphipathicity, helicity, or hydrophobicity. For instance, substituting leucine (Leu) 9 and 13 in melittin with the less hydrophobic residue Ala resulted in decreased activity toward *S. aureus* and *B. subtilis* (Pandey et al., 2010). Interestingly, however, the substituted melittin maintained its activity toward *E. coli* and was still active when only Leu9 or only Leu16, or Leu9 and Leu16 were substituted with Ala. However, when Leu6, Leu13, and isoleucine (Ile) 20 of melittin were substituted with the peptoid (residues with the side-chain connected to the backbone nitrogen rather than the α-carbon) versions of Ala, Leu, Phe, and Lys, antimicrobial activity was reduced by 2- to 10-fold (Liu et al., 2007). These structural substitutions affected both the helicity and the overall hydrophobicity of melittin, which in turn affected its activity. In this study, Lys was substituted with analogs that differed from each other solely by the length of their sidechain. This did not affect the overall charge of the peptide and is unlikely to have affected hydrophobicity, as all peptides exhibited the same retention time in RP-HPLC. Previous studies where Lys has been substituted with these same residues have reported little to no effect on helicity but reduced AMP toxicity to mammalian cells (Khara et al., 2016; Kohn et al., 2018; Mant et al., 2019). Despite the limited change reported in the activity of the previously mentioned peptides (magainin II, D16, C18G, and tritrpticin) where Lys had also been replaced with Orn, Dab, and Dap, reduced toxicity was observed in these analogs, indicating that the major advantage of lysine substitution with Orn, Dab, and Dap is reducing toxicity but not altering helicity or substantially improving antimicrobial activity.

### Cytotoxicity of melittin, melittin dimers, and the Lys-substituted melittin analogs

The ability of melittin, melittin dimers, and the Lys-substituted melittin analogs to lyse eukaryotic cells and inhibit proliferation was determined using H4IIE cells and HEK293 cells. Percent lysis and percent proliferation for each peptide against each cell type indicate that each peptide is highly cytotoxic, as indicated by the LD_50_ and IC_50_ (Table 4).

**TABLE 4:** LD_50_, IC_50_, and HC_50_ of melittin, melittin dimers, and Lys-substituted melittin toward H4IIE and HEK293 cells and chicken red blood cells (RBCs), respectively.

	H4IIE		HEK293		RBCs
Peptide	LD_50_ (µM)	IC_50_ (µM)	LD_50_ (µM)	IC_50_ (µM)	HC_50_ (µM)
1. Melittin	1.79 ± 0.95	2.88 ± 0.08	0.75 ± 0.21	9.47 ± 3.63	0.59 ± 0.04
2. Lys → Orn melittin	2.22 ± 1.31	3.37 ± 0.08	1.16 ± 0.09	10.49 ± 2.65	1.4 ± 1.14
3. Lys → Dab melittin	2.42 ± 1.82	3.08 ± 0.24	1.19 ± 0.22	9.23 ± 2.91	1.33 ± 1.02
4. Lys → Dap melittin	5.43 ± 0.91	7.93 ± 0.12	2.02 ± 0.77	14.32 ± 6.02	0.89 ± 0.39
5. Parallel dimer	1.13 ± 0.35	2.44 ± 0.78	0.63 ± 0.18	5.04 ± 3.05	0.20 ± 0.03
6. Antiparallel dimer	4.66 ± 4.49	7.91 ± 0.82	2.01 ± 0.66	12.96 ± 1.91	0.29 ± 0.02

Another measure of cytotoxicity is hemolysis of red blood cells. Here, hemolysis toward chicken red blood cells was determined using standard protocols in our laboratory (Lam et al., 2016). The HC_50_ is shown in Table 4, with the low concentration of each peptide (∼3× less the antimicrobial activity of each peptide), indicating the extreme hemolytic nature of melittin and its analogs.

The LD_50_ and IC_50_ for each cell line, as well as the HC_50_, were used to calculate the therapeutic index (TI), which is a measure of a material’s biological safety (Table 5). Generally, the higher the therapeutic index, the safer and less toxic a material is, with a TI of 10 being a threshold point between a material being considered safe (TI greater than 10) or toxic (TI less than 10) (Tamargo et al., 2015). The TIs of melittin and each of its analogs, based on cell lysis and hemolysis and the MIC_50_/MBC_50_ toward *S. aureus* (Table 5), were very low (<10), indicating that these peptides are extremely toxic and would not be suitable for therapeutic use as antimicrobials. When considering the TI based on the IC_50_, peptide 4 (Dap-melittin) was the least toxic, with a TI of 18.07 (based on its IC_50_ for HEK293 cells). Overall, although still considered poor TIs, peptides 2, 3, and 4 (Orn-, Dab-, and Dap-melittin) had higher TIs than peptide 1 (native melittin), and both peptides 5 and 6 (parallel and antiparallel dimers) had lower TIs. When the TIs were calculated using the MIC_50_/MBC_50_ toward *E. coli* (Supplementary Table S1), none of the peptides exhibited TIs >10. However, in general, peptides 2–6 all had higher TIs than native melittin.

**TABLE 5:** Therapeutic index for cell lysis, cell proliferation, and hemolysis of H4IIE and HEK293 cells based on the antimicrobial activity of the peptides toward *S. aureus*.

	H4IIE		HEK293		
Peptide	TI (cell lysis)^a^	TI (cell proliferation)^a^	TI (cell lysis)^a^	TI (cell proliferation)^a^	TI (hemolysis)^a^
1. Melittin	2.05	4.01	0.85	13.18	0.67
2. Lys → Orn melittin	2.80	5.26	1.47	16.39	1.80
3. Lys → Dab melittin	2.74	4.21	1.35	12.60	1.50
4. Lys → Dap melittin	3.93	10.00	1.47	18.07	0.64
5. Parallel dimer	0.36	1.27	0.20	2.63	0.06
6. Antiparallel dimer	1.28	3.06	0.55	5.01	0.08

^a^ Therapeutic index (TI) determined for cell lysis by LD_50_/MBC_50_, for inhibition of proliferation by IC_50_/MIC_50_, and hemolysis by HC_50_/MBC_50_ (MIC_50_ and MBC_50_ of peptides toward *S. aureus* 29213 used to calculate TI).

All peptides were generally far more lytic toward HEK293 cells than H4IIE cells. Peptides 1, 2, 3, and 6 were the most toxic, and peptides 4 and 5 were the least toxic; however, the differences were not considered significant at peptide concentrations relative to their MBC values. Previous reports of substituting Lys with Orn, Dab, or Dap have shown that each shorter iteration is less and less toxic, and although similar trends were observed here (LD_50_/IC_50_ peptide 4 > peptide 3 > peptide 2), the differences were not considered significant, and peptide 4 was more hemolytic than peptides 2 and 3 (O’Brien-Simpson et al., 2016; Mant et al., 2019). Similarly, for hemolysis at concentrations within the MBC range of these peptides, melittin and all analogs were equally very lytic. Although the substitutions and dimerization improved melittin’s activity toward *E. coli*, the concentrations at which the analogs were active were greater than for *S. aureus* and were thus still very toxic. Better TIs were achieved using the MIC and MBC values of melittin and analogs toward *S. aureus* than for *E. coli*.

The lack of change in activity for the Lys-substituted melittin toward *S. aureus* supports the theory that the positively charged amino acids are predominantly involved in the initial electrostatic interaction with the bacterial membrane. The length of the side chains on the polar face of an AMP has been shown to be a determining factor in the degree of insertion of the peptide into the lipid membrane, with shorter side chains resulting in the peptide being less deeply inserted into the membrane (Uggerhøj et al., 2015; Zelezetsky et al., 2005). This is thought to be due to the shorter alkyl groups increasing the polarity. This does not always result in better activity or reduced toxicity, as shown here and in several other studies, but a decrease in activity is rarely observed (Lu et al., 2020). One advantage of Lys substitutions with Orn, Dab, and Dap is that these substitutions decrease the susceptibility of peptides to protease degradation, thereby increasing the half-life of the peptides (Arias et al., 2018; Khara et al., 2016; Lu et al., 2020). Thus, although the Lys substitutions or dimerization did not always alter antimicrobial activity or significantly reduce the cytotoxicity of melittin in this study, they remain valuable tools in the chemistry toolbox for altering the activity of antimicrobial peptides.

## Conclusion

This work explores the extent to which AMPs can tolerate structural changes and the effect of lysine substitution and dimerization on melittin. Whilst dimerization of the AMP melittin did not result in significantly improved antimicrobial activity toward *S. aureus* or reduced toxicity at antimicrobial concentrations, this result does not indicate that this strategy would not benefit other AMPs or other microbes. As shown here, dimerization and Dab and Dap substitution significantly improved the antibacterial activity toward *E. coli*. Given the improved activity observed in these analogs toward *E. coli*, testing them in other Gram-positive and Gram-negative bacteria would be of interest to determine whether the improved activity is strain specific. As for toxicity, melittin is already an extremely lytic AMP with no specificity for any organism, meaning reducing toxicity rather than improving its activity may be of more consequence. The non-proteinogenic amino acids Orn, Dab, and Dap were explored as alternatives to Lys in the sequence of melittin to try and address the issue of toxicity. Although unsuccessful in significantly decreasing toxicity at antimicrobial concentrations, peptides 4 (Dap-melittin) and 6 (antiparallel melittin) did show decreased toxicity compared to peptide 1 (melittin) at sub-inhibitory concentrations. It was also shown that these amino acid substitutions did not negatively impact activity or toxicity and, in some cases, even improved activity. This shows these alterations may still have a role to play in the rational design and modification of other AMPs and in other clinical uses for melittin, such as cancer therapies. Numerous studies investigating the anticancer effects of melittin have found it to be effective at doses lower than the MBCs observed in this study (Mahmoodzadeh et al., 2015; Chang et al., 2021; Mir Hassani et al., 2021; Duffy et al., 2020; Jeong et al., 2014). Observing the effect of substituting Lys with Orn, Dab, and Dap in other AMPs would help further our understanding of the role these cationic residues play in membrane insertion, activity, and toxicity. Changing only one Lys, or combinations thereof, would also help explore how specific sequence position impacts their role in peptide structure and activity. Lys has been shown to be important for peptide activity (Torres et al., 2019), with decreased antimicrobial activity observed when substituted with ε-lysine, but no change in activity was observed when Lys^21^ and Lys^23^ were substituted (Mayandi et al., 2020). Additionally, this could be attempted for the positively charged Arg within the melittin sequence. Given that substituting Lys with Arg often increases activity as well as toxicity, it would be worthwhile investigating whether short-chain analogs of Arg can maintain increased activity but reduce toxicity. In a study by Oren and Shai (1997), Lys (Wayne, 2009), Val (Zasloff, 2002; Askari et al., 2021), and Ile (O’Brien-Simpson et al., 2016) were replaced with D-amino acid equivalents, resulting in a loss of helicity, reduced toxicity but a retention in antimicrobial activity. However, in another study, D-amino acid melittin single peptide was found to retain its toxicity and antimicrobial activity (Sylvestre et al., 2021), whereas a polymer form of D-melittin has significantly reduced toxicity. In future studies, determining the conformation of these analogs would also be useful for establishing the effect these substitutions and dimerization or oligo/polymerization have on the structure and whether a lack of conformational flexibility alters their activity. This study has strongly highlighted melittin’s toxicity as its greatest impediment to potential clinical development, as have many others (Askari et al., 2021; Memariani et al., 2019; Guha et al., 2021; Erkoc et al., 2022; Rady et al., 2017; Haque et al., 2023). However, encapsulation in nanocarriers (predominantly being researched for melittin’s anticancer effects), truncated versions of melittin, conjugation to other substances, and synergistic effects of melittin with antibiotics all have the potential to reduce the effective dose of melittin to non-toxic levels (Ye et al., 2021; Gui et al., 2020; Subbalakshmi et al., 1999; Nabizadeh et al., 2023; Bardbari et al., 2018). The topical application remains a potential clinical pathway for full-length melittin, but future studies may benefit from making more drastic changes to melittin rather than single amino acid substitutions or dimerization alone (An et al., 2018; Lima et al., 2021; Bae et al., 2022).

## Data Availability

The raw data supporting the conclusions of this article will be made available by the authors, without undue reservation.
