# Supplementary material for: Dimerization and lysine substitution of melittin have differing effects on bacteria
Source: Front Pharmacol. 2024 Oct 7;15:1443497. doi: 10.3389/fphar.2024.1443497 (PMC11492869; doi:10.3389/fphar.2024.1443497)
Supplement: Supplementary file 1 [file DataSheet1.PDF]

Supplementary figures

A

B

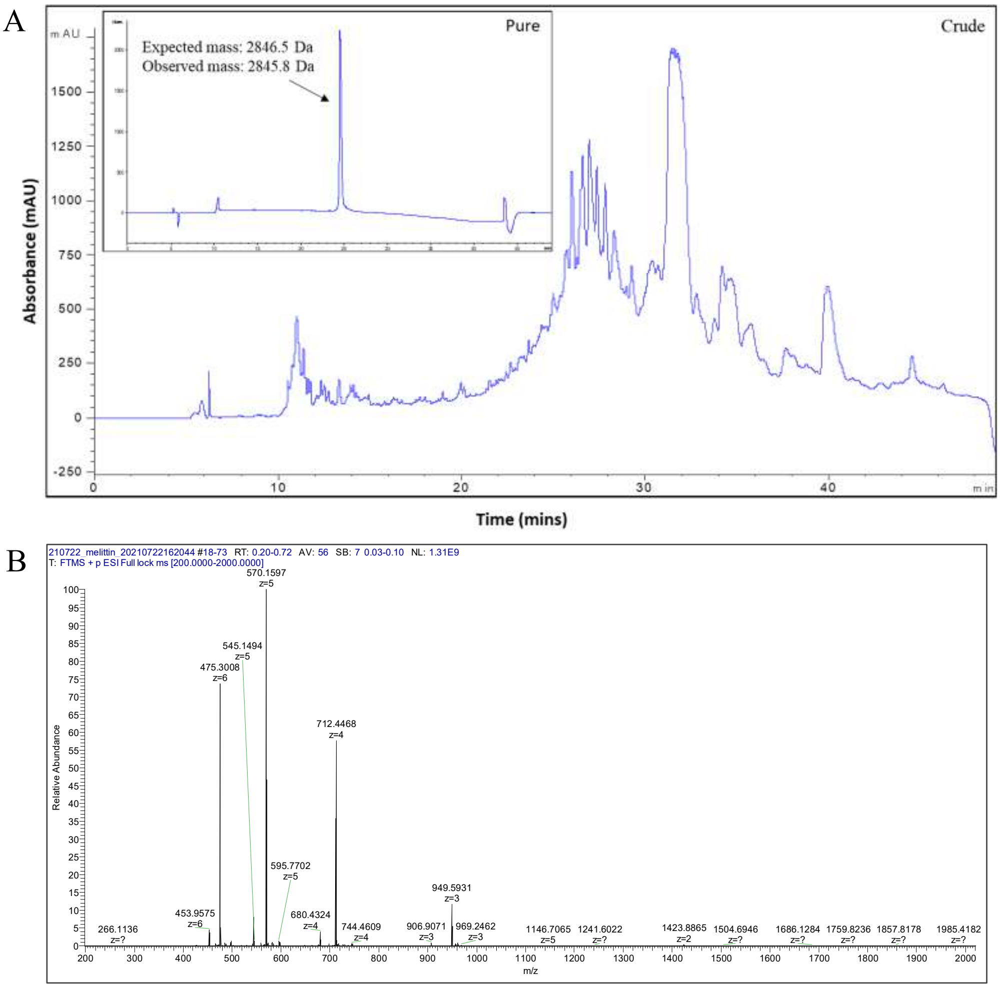

Figure 1. RP-HPLC profile of crude melittin and the purified product (> 99% purity).

(A) RP-HPLC profile of crude melittin peptide with the corresponding RP-HPLC of purified melittin as well as the expected and observed mass. The crude peptide was purified using a semi-preparative ZORBAX 300 SB-C18 column installed in a HPLC 1200 system under a flow rate of 2 ml/min using buffer A (0.1% v/v TFA in milliQ water) and buffer B (0.1%TFA in 90% acetonitrile, 10% milliQ water, v/v) as the limiting solvent. Peptide detection was performed by absorbance at 214 nm. (B) The peptide mass confirmed using an Exactive™ OrbiTrap Mass Spectrometer.

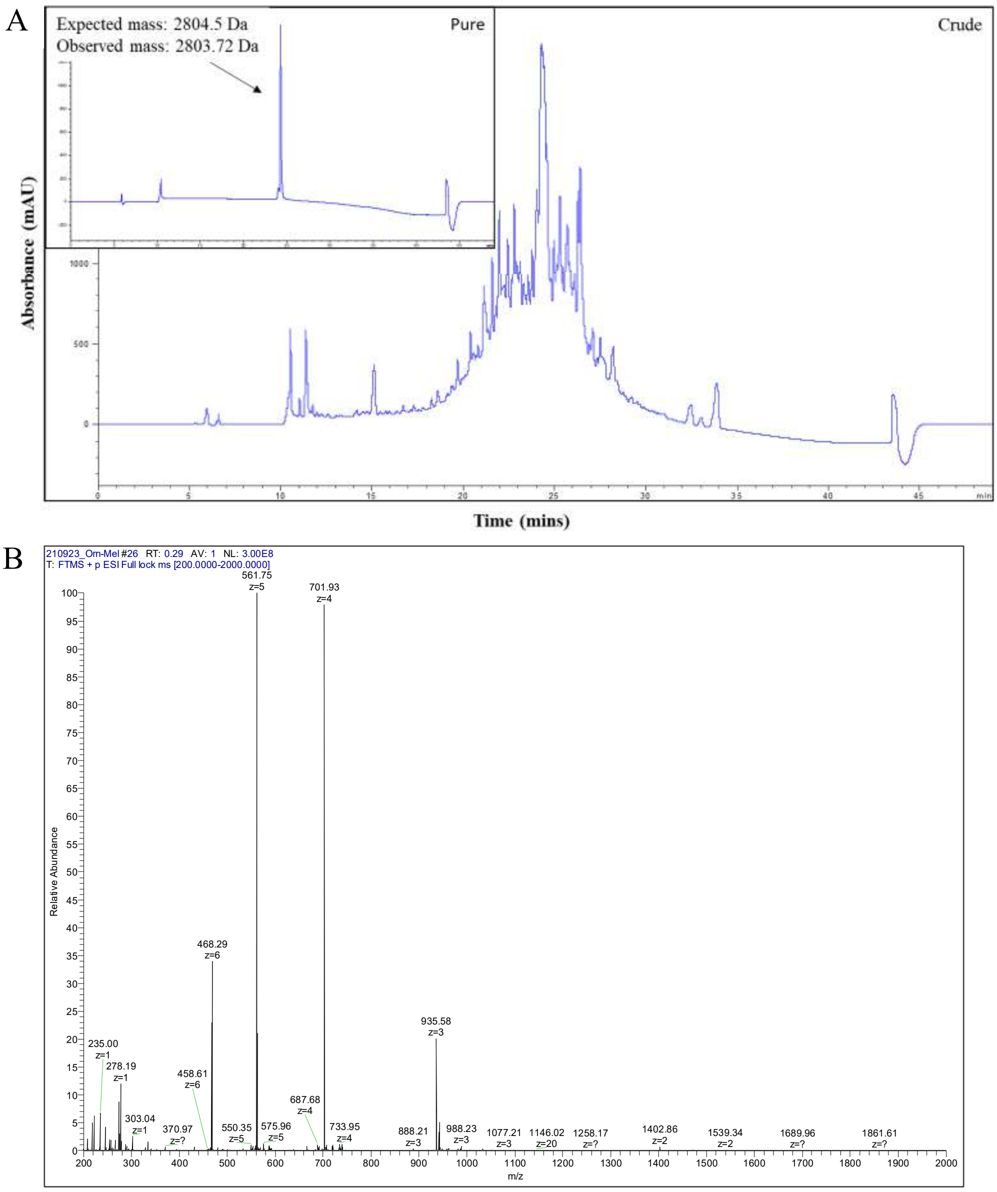

Figure 2. RP-HPLC profile of crude Lys → Orn melittin and the purified product (> 95% purity).

(A) RP-HPLC profile of crude Lys → Orn melittin with the corresponding RP-HPLC of purified melittin as well as the expected and observed mass. The crude peptide was purified using a semi-preparative ZORBAX 300 SB-C18 column installed in a HPLC 1200 system under a flow rate of 2 ml/min using buffer A (0.1% v/v TFA in milliQ water) and buffer B (0.1%TFA in 90% acetonitrile, 10% milliQ water, v/v) as the limiting solvent. Peptide detection was performed by absorbance at 214 nm. (B) The peptide mass confirmed using an Exactive™ OrbiTrap Mass Spectrometer.

A

B

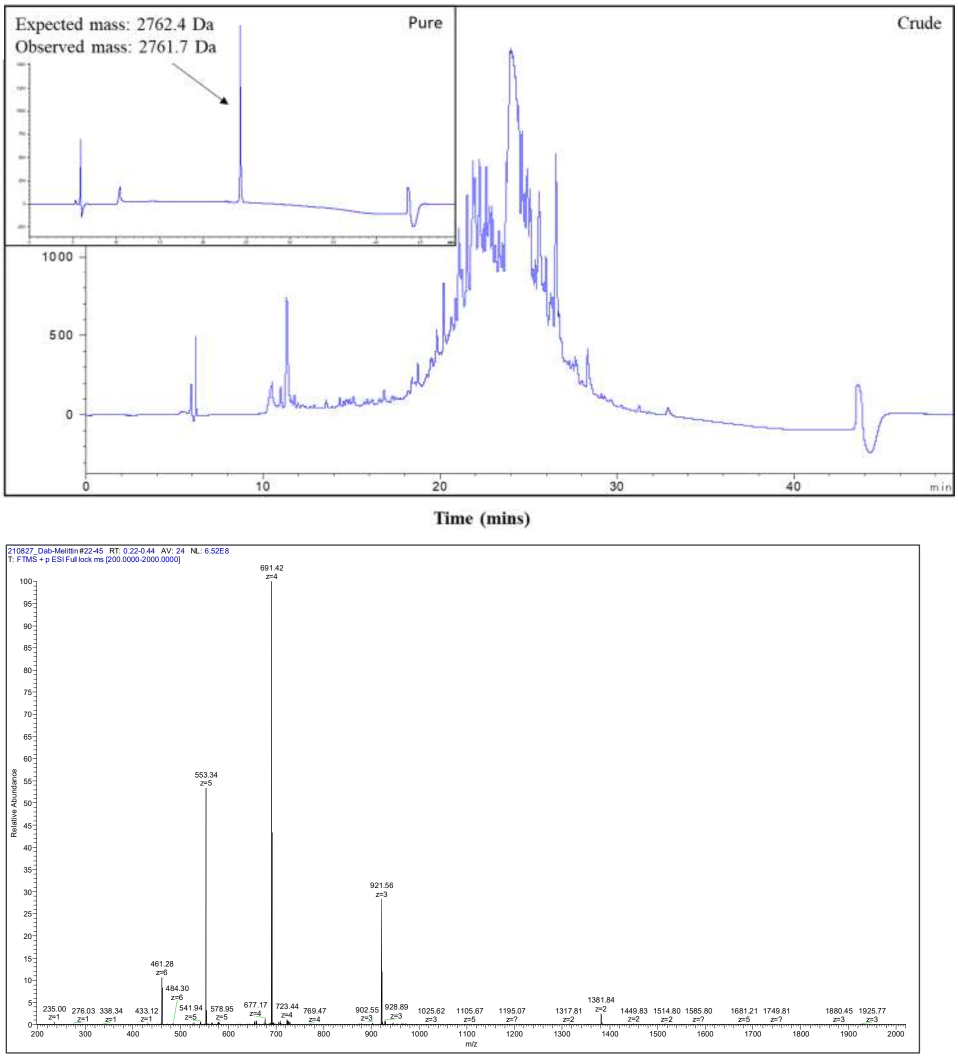

Figure 3. RP-HPLC profile of crude Lys → Dab melittin and the purified product (> 99% purity). (B) The peptide mass confirmed using an Exactive™ OrbiTrap Mass Spectrometer.

RP-HPLC profile of crude Lys → Dab melittin with the corresponding RP-HPLC of purified melittin as well as the expected and observed mass. The crude peptide was purified using a semi-preparative ZORBAX 300 SB-C18 column installed in a HPLC 1200 system under a flow rate of 2 ml/min using buffer A (0.1% v/v TFA in milliQ water) and buffer B (0.1%TFA in 90% acetonitrile, 10% milliQ water, v/v) as the limiting solvent. Peptide detection was performed by absorbance at 214 nm. (B) The peptide mass confirmed using an Exactive™ OrbiTrap Mass Spectrometer.

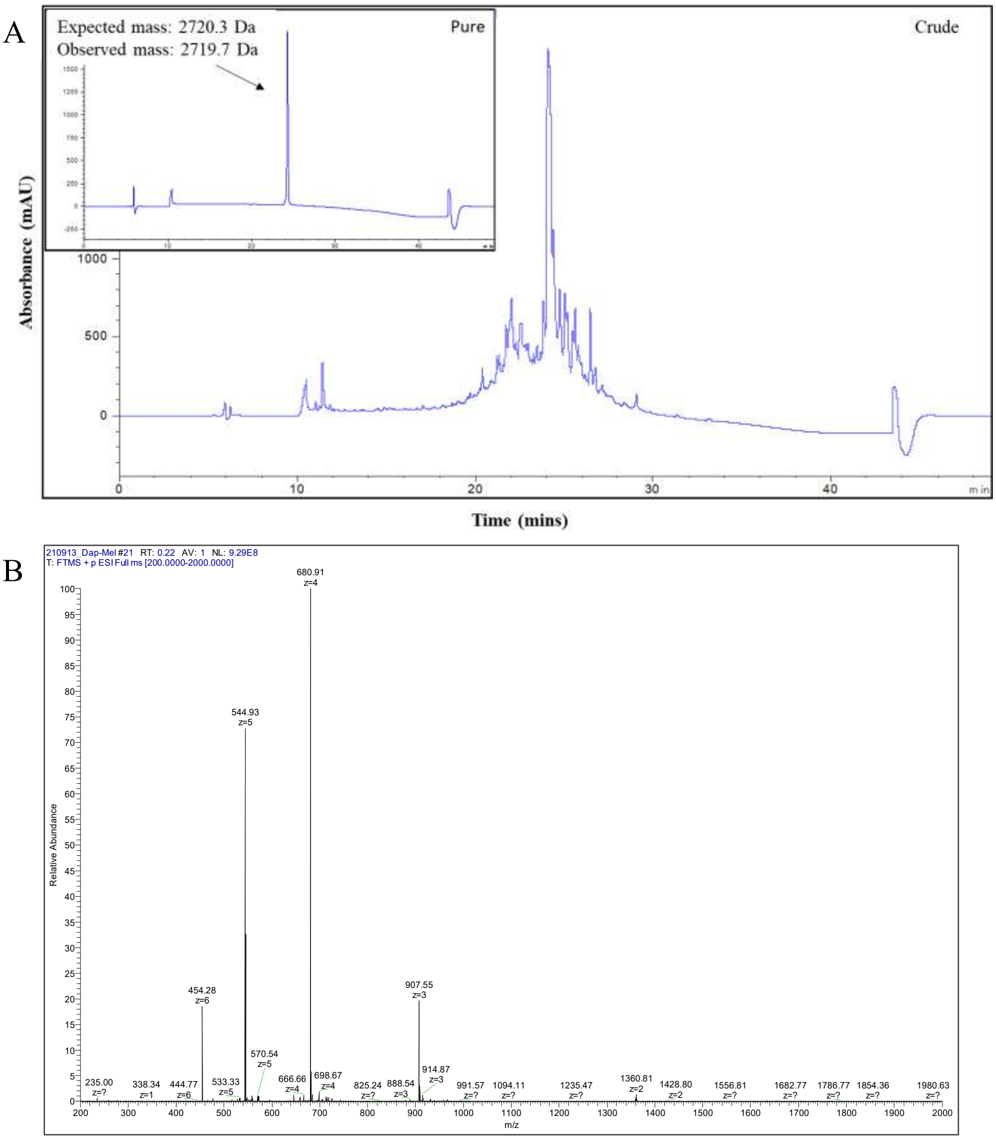

Figure 4. RP-HPLC profile of crude Lys → Dap melittin and the purified product (> 99% purity). (B) The peptide mass confirmed using an Exactive™ OrbiTrap Mass Spectrometer.

RP-HPLC profile of crude Lys → Dap melittin with the corresponding RP-HPLC of purified melittin as well as the expected and observed mass. The crude peptide was purified using a semi-preparative ZORBAX 300 SB-C18 column installed in a HPLC 1200 system under a flow rate of 2 ml/min using buffer A (0.1% v/v TFA in milliQ water) and buffer B (0.1%TFA in 90% acetonitrile, 10% milliQ water, v/v) as the limiting solvent. Peptide detection was performed by absorbance at 214 nm. (B) The peptide mass confirmed using an Exactive™ OrbiTrap Mass Spectrometer.

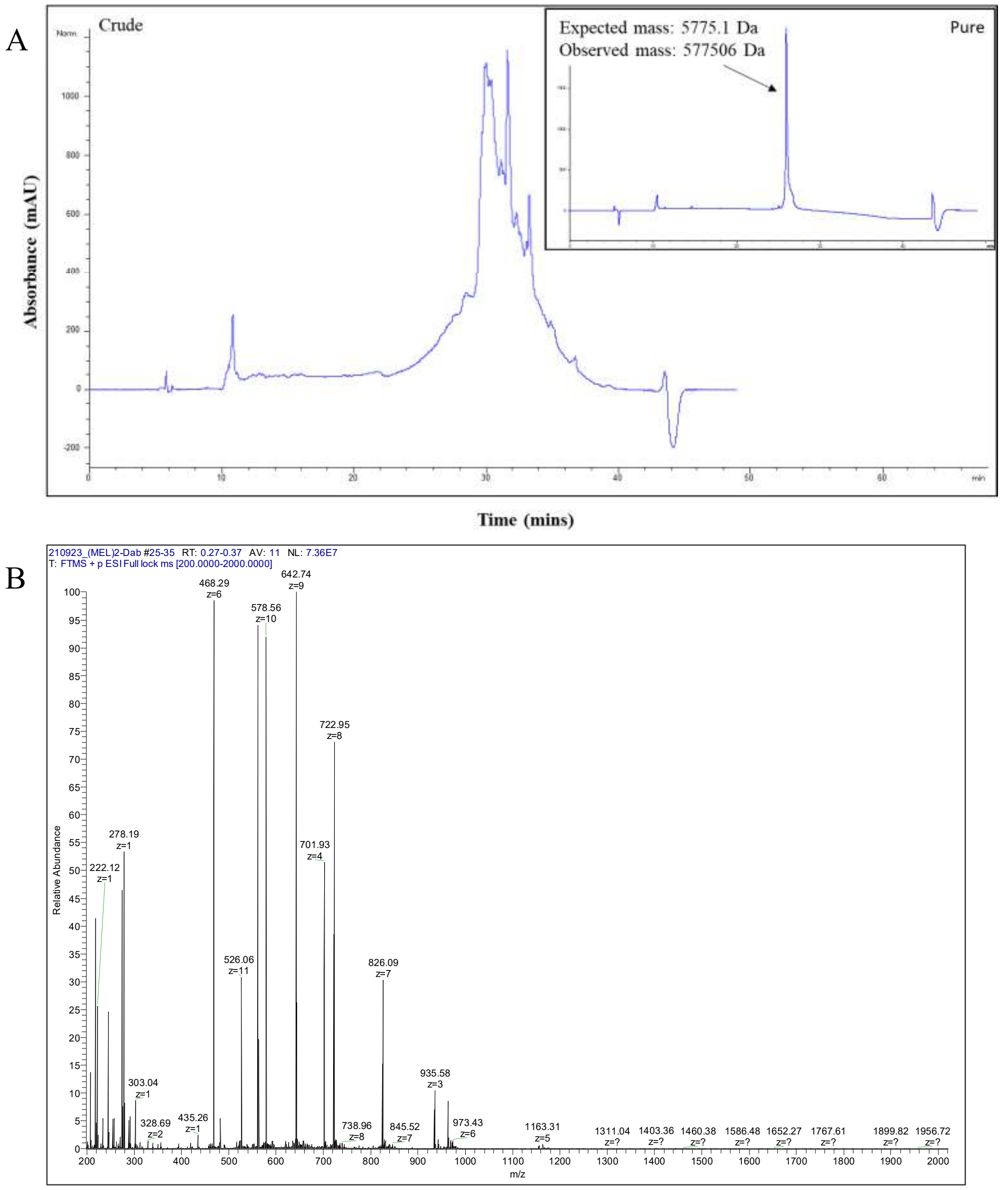

Figure 5. RP-HPLC profile of crude parallel melittin dimer and the purified product (> 90% purity).

RP-HPLC profile of crude parallel melittin dimer with the corresponding RP-HPLC of purified melittin as well as the expected and observed mass. The crude peptide was purified using a semi-preparative ZORBAX 300 SB-C18 column installed in a HPLC 1200 system under a flow rate of 2 ml/min using buffer A (0.1% v/v TFA in milliQ water) and buffer B (0.1%TFA in 90% acetonitrile, 10% milliQ water, v/v) as the limiting solvent. Peptide detection was performed by absorbance at 214 nm. (B) The peptide mass confirmed using an Exactive™ OrbiTrap Mass Spectrometer.

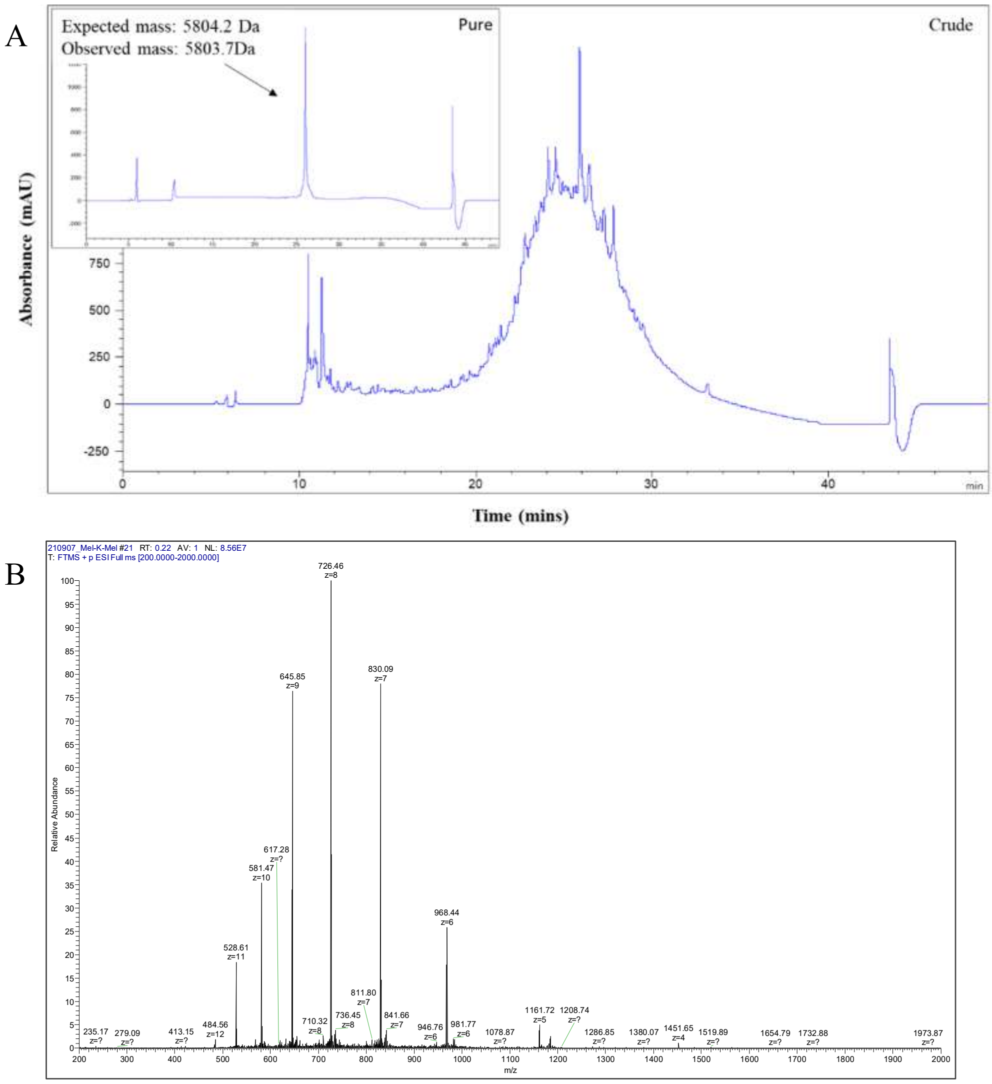

Figure 6. RP-HPLC profile of crude antiparallel melittin dimer and the purified product (> 90% purity).

RP-HPLC profile of crude antiparallel melittin dimer with the corresponding RP-HPLC of purified melittin as well as the expected and observed mass. The crude peptide was purified using a semi-preparative ZORBAX 300 SB-C18 column installed in a HPLC 1200 system under a flow rate of 2 ml/min using buffer A (0.1% v/v TFA in milliQ water) and buffer B (0.1%TFA in 90% acetonitrile, 10%milliQ water, v/v) as the limiting solvent. Peptide detection was performed by absorbance at 214 nm. (B) The peptide mass confirmed using an Exactive™ OrbiTrap Mass Spectrometer.

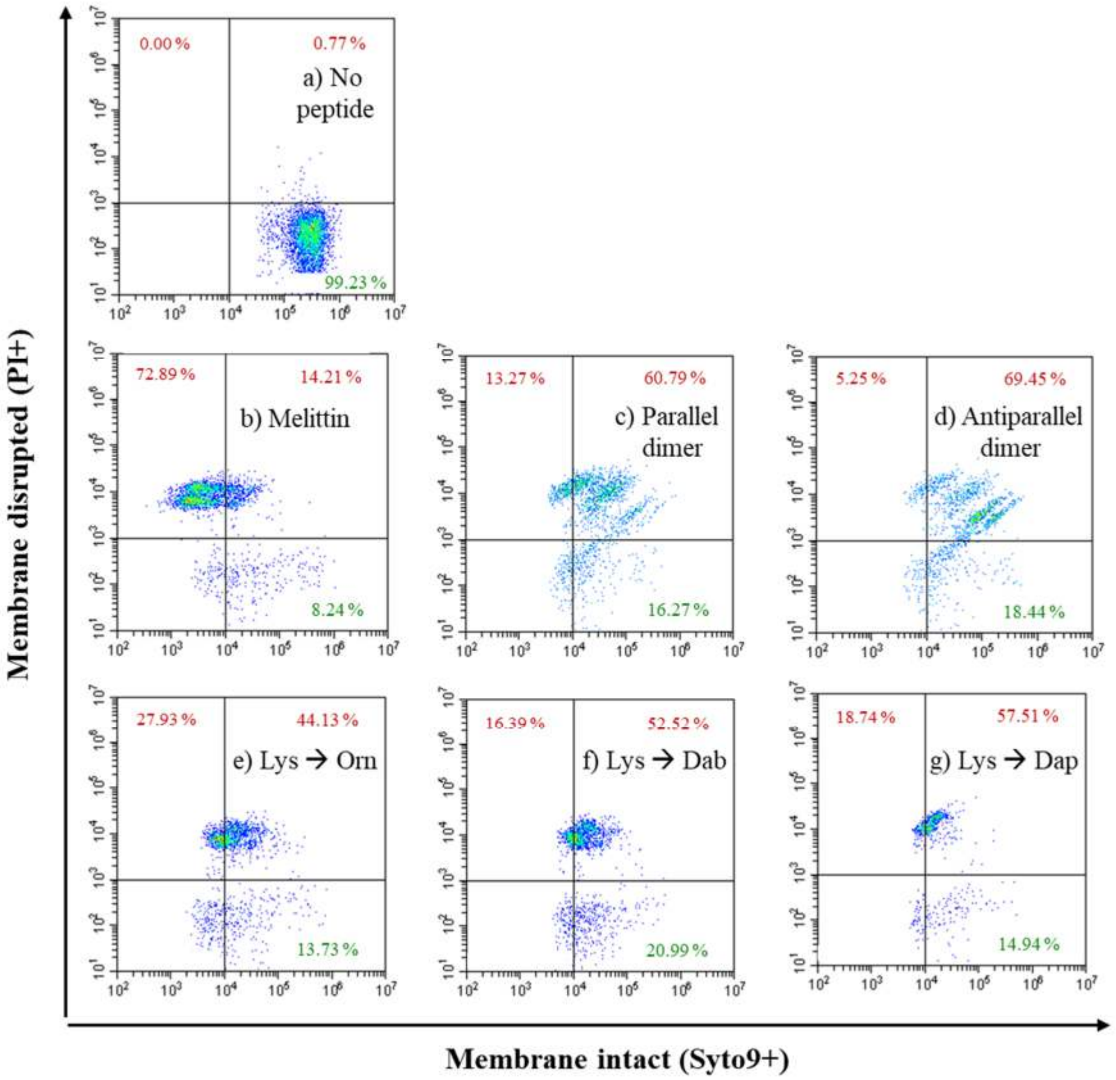

Figure 7. Membrane disruption of S. aureus caused by ~0.5 x MBC of melittin, melittin dimers and Lys-substituted melittin.

At ~0.5 x MBC of melittin (b), melittin dimers (c and d) and all Lys-substituted melittin (e, f, and g), membrane permeabilisation of *S. aureus* was observed on the flow cytometer. Dimerisation and Lys substitution exhibited the same level of permeabilisation as melittin. However, the parallel (c) and antiparallel (d) dimers created a striated appearance among the PI+ cells.

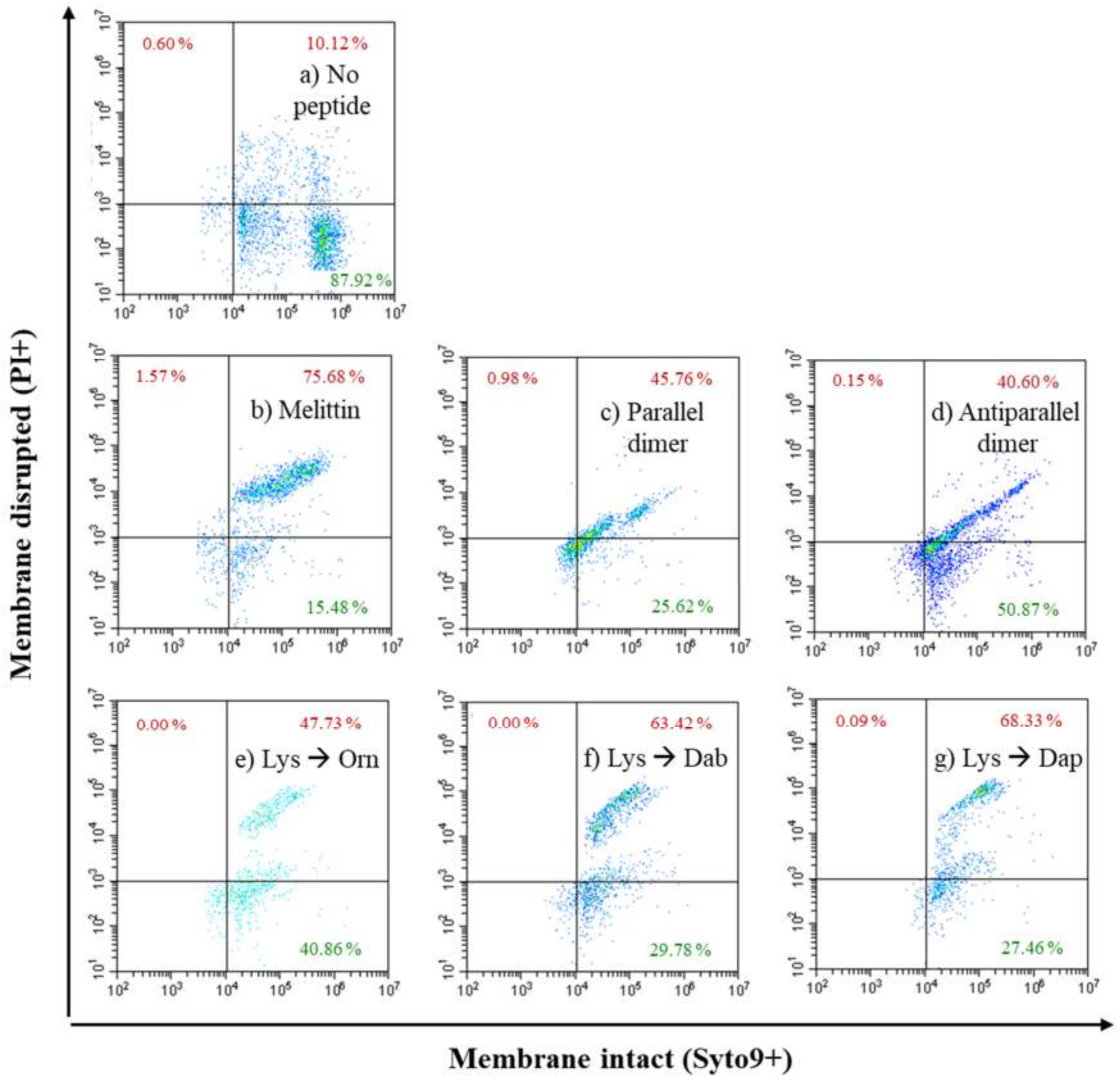

Figure 8. Membrane disruption of E. coli caused at ~0.5 x MBC of melittin, melittin dimers and Lys-substituted melittin.

At ~0.5 x MBC of melittin (b), melittin dimers (c and d) and all Lys-substituted melittin (e, f, and g), membrane permeabilisation of *E. coli* was observed on the flow cytometer. Dimerisation and Lys substitution exhibit a significantly reduced level of permeabilisation compared to melittin.

Table 1. Therapeutic index for cell lysis, cell proliferation and haemolysis of H4IIE and HEK293 cells based on the antimicrobial activity of the peptides towards E. coli.

	H4IIE		HEK293		
	TI (Cell lysis)[a]	TI (Cell proliferation)[a]	TI (Cell lysis)[a]	TI (Cell proliferation)[a]	TI (haemolysis)[a]
**Melittin**	0.41	0.76	0.17	2.49	0.14
**Parallel dimer**	0.51	1.08	0.28	2.23	0.09
**Antiparallel dimer**	1.65	2.63	0.71	4.31	0.10
**Lys → Orn melittin**	0.63	1.56	0.33	4.86	0.40
**Lys → Dab melittin**	0.86	1.12	0.42	3.35	0.47
**Lys → Dap melittin**	1.93	2.82	0.72	5.10	0.32

a - Therapeutic index (TI) determined for cell lysis by LD_{50}/MBC_{50}, for inhibition of proliferation by IC_{50}/MIC_{50} and haemolysis by HC_{50}/MBC_{50} (MIC_{50} and MBC_{50} of peptides towards *E. coli* 8729 used to calculate TI)
